# Removing mercury, protecting people’s health

**DOI:** 10.2471/BLT.18.020118

**Published:** 2018-01-01

## Abstract

Now that the Minamata Convention has come into effect, developing countries are struggling to phase out the use of mercury to protect people’s health. Jan Dirk Herberman reports.

Every day children who work alongside adults in Ghana’s small-scale mining sector are risking their lives without realizing it.

“Children are involved in small-scale gold mining in many ways,” says Edith Clarke, Head of the Programme on Environmental and Occupational Health at the Ghana Health Service in Accra.

“They get ore from gold mines and together with the mercury, they bring it to their homes, where they use the mercury to extract gold from the ore,” Clarke says, adding that under a 1998 national law, children should be protected from such exploitative child labour – defined as work that deprives a child of its health, education or development.

To extract the precious metal, the mercury is heated in a pan on an open fire, poured over the ore and eventually the mercury separates the gold from the other substances.

This rudimentary practice – known as artisanal and small-scale gold mining – is a major source of income for many families in Ghana.

But, because of the widespread use of mercury – especially inhalation of mercury vapours during the refining process – and other occupational and environmental risks associated with this mining sector, this form of gold mining is risky.

“Mercury is toxic for human health,” explains Carolyn Vickers, team leader for chemical safety at the World Health Organization (WHO). “It poses a particular threat to development *in utero* and in early life.”

Mercury, an element also known as quicksilver, exists in several forms, each with different toxic effects, including on the nervous, digestive and immune systems, as well as the lungs, kidneys, skin and eyes. Exposure to it can result in severe illness and death.

“While focused primarily on mercury, the Convention also provides an important platform for multisectoral engagement to address other health issues.”

Carolyn Vickers

The Minamata Convention on Mercury was established by the international community to protect people and the environment from mercury use.

The agreement is named after a Japanese city where a chemical factory released large quantities of mercury into Minamata Bay contaminating the water and killing the fish.

As a result of eating contaminated fish, about 900 people died and more than 2000 people suffered the effects of mercury poisoning – Minamata disease – over more than three decades.

The Minamata Convention, which calls for the phasing out of the use of mercury from several sectors, was adopted in 2013 and entered into force this year.

So far 84 countries have ratified the treaty, which prohibits new mercury mines, requires countries to phase out existing mines and bans the manufacture, import and export of mercury in many products.

Some of these products are in the health sector and include sphygmomanometers, for measuring blood pressure, and mercury thermometers.

An important exception is thiomersal (ethyl-mercury), which is used in some human and animal vaccines, as there is no evidence that the amount of thiomersal used in vaccines poses a health risk.

Parties to the convention – the countries that have ratified the treaty – are expected to draw up plans to reduce and, if possible, eliminate the use of mercury in small-scale gold mining and set thresholds for emissions of mercury from industrial sources, including coal-fired power plants.

“While focused primarily on mercury, the Convention also provides an important platform for multisectoral engagement to address other health issues, for example, those affecting small-scale gold miners and their communities,” Vickers says.

But, the convention presents challenges, particularly for developing countries. “It is quite complex to implement and this will take time,” Clarke says.

For Siriwan Chandanachulaka, Director of the Environmental Health Bureau in the Ministry of Public Health in Thailand, there are three main challenges for implementation: the large number of sectors concerned, the many stakeholders involved and the high cost of replacing mercury.

“We need to phase out mercury-added products – such as thermometers and sphygmomanometers, as well as amalgam tooth fillings as they also contain mercury – that have been used widely in hospitals and clinics for many years,” she says.

Before replacement gets under way, health workers across Thailand will need to do a situation assessment and inventory, including an estimate of the cost of replacing these instruments and materials.

WHO provides guidance on the replacement process, recommending a step-by-step approach.

“This will be easy to follow,” says Siriwan, “but first we need the support of the health sector to get started.”

“We need to talk to the medical staff, medical associations, universities and private companies that are operating health facilities to convince them of the need to phase out these familiar instruments and replace them with digital ones, perhaps through media campaigns and hospital brochures,” Siriwan says.

Dr Thahirahtul Asma Zakaria is leading the work of Malaysia’s Ministry of Health on an implementation plan for the health aspects of the Convention, which means replacing mercury thermometers and sphygmomanometers with their digital equivalents.

“The implementation is a real team effort for which full cooperation from all divisions and all affected people in government, the economy and the public health sector is needed,” Zakaria says.

To understand the challenges facing the health sector and to plot out the adjustments that need to be made, the health ministry in Malaysia conducted a workshop for managers in the health sector with WHO support.

Another challenge for Malaysia’s health sector is to identify and protect people who may have been exposed to mercury among Malaysian population.

Ghana is also in the early stages of implementing the Minamata Convention and – given the significant health burden in small-scale gold mining – the country’s priority is to stop the use of mercury in this sector.

Known as the Gold Coast in colonial times, Ghana is one of the world’s top 10 gold producers.

While big, established companies – accounting for about 65% of the gold mining sector – use sophisticated tools and processes to extract the precious metal, people in artisanal and small mines are still exposed to mercury by handling it directly.

Between 500 000 and one million artisanal and small-scale miners are operating in Ghana. Only 20–30% of them work in licensed mines that are subject to regulatory controls. The rest work in the informal sector, where conditions are not inspected for health and safety standards.

Ghana’s government has tried for a long time to crack down on the informal sector – with arrests, closure of mines, confiscation of excavators and other equipment – with little success. The latest attempt was a six-month moratorium on the entire artisanal and small-scale mining sector, both legal and illegal, that was extended until the end of last year.

“Many of the artisanal and small-scale mines do not have licenses,” explains Edith Clarke. “We had to issue a blanket ban on small-scale gold mining, as it was the only way to implement Minamata and protect the health of the people in the industry,” Clarke says.

The moratorium – where it has been observed – has caused widespread anger among the workers, many of whom have lost their main source of income.

To provide an alternative source of income, the government has launched the Multilateral Mining Integrated project, under which previously illegal miners can work in established mines and use centralized plants that do not use mercury to process the ore.

Under the five-year project, mining companies cede parts of their mining concessions to the government. The process of transfer of partial concessions began last year, Clarke says.

“Our government is trying to streamline arrangements for miners, so that they engage in more sustainable forms of small-scale mining that do not endanger the environment. We are particularly keen to reduce pollution of water bodies,” Clarke says.

Ghana’s government is also concerned about the long-term health effects on people working in the industry.

“It‘s difficult for us to identify the victims of mercury poisoning.”

Edith Clarke

“It‘s difficult for us to identify the victims of mercury poisoning,” Clarke says, explaining that Ghana lacks the diagnostic facilities and laboratories to detect Minamata disease, so that people who need it can receive treatment to remove the metal from their bodies.

Ghana, Malaysia, Thailand and many other countries have not yet done their assessments or estimated the costs of replacing mercury. Some countries are expected to seek international support for this step.

“The Convention mentions financial support, technical assistance and technology transfer to countries with economies in transition and developing countries,” Siriwan explains.

Two international funds are earmarked for this purpose: the Global Environment Facility Trust Fund, set up in 1992, which is already supporting mercury projects in countries, and an international programme to be set up by United Nations Environment.

Without vital international support, many implementing countries in Africa and Asia could run into severe difficulties, says Clark.

“The cost of phasing out the use of mercury could be a reason why some African countries are hesitating to join the convention,” she says.

Her own country, Ghana, could pave the way for other countries in the region to follow suit. “If we deliver and show how it can be done, other countries in Africa may also be more motivated to sign up to the Minamata Convention.”

**Figure:**
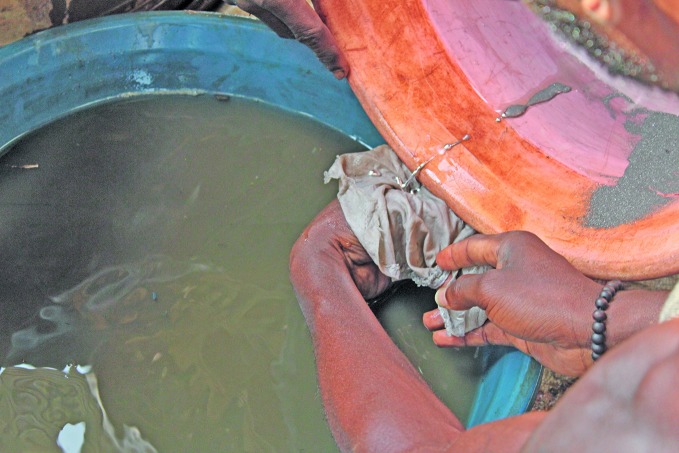
Liquid mercury is added to gold ore in a small-scale gold mine in Ghana.

WHO/Michaela Pfeiffer

**Figure:**
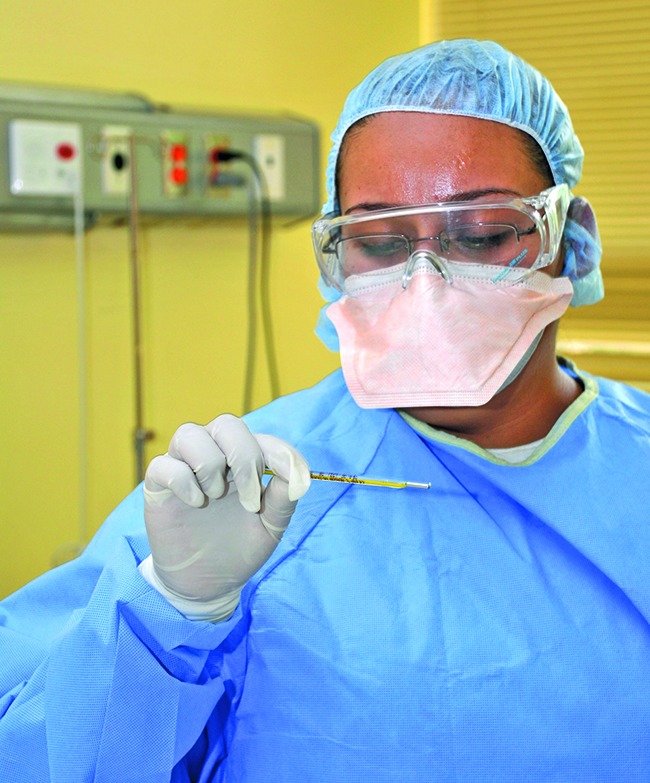
A health worker uses a mercury thermometer during the epidemic of influenza A H1N1 in Mexico in 2009.

WHO /Harold Ruiz

